# Differentiation of Glioma Mimicking Encephalitis and Encephalitis Using Multiparametric MR-Based Deep Learning

**DOI:** 10.3389/fonc.2021.639062

**Published:** 2021-03-15

**Authors:** Wenli Wu, Jiewen Li, Junyong Ye, Qi Wang, Wentao Zhang, Shengsheng Xu

**Affiliations:** ^1^ Department of Radiology, The First Affiliated Hospital of Chongqing Medical University, Chongqing, China; ^2^ Key Laboratory of Optoelectronic Technology and Systems of the Ministry of Education, Chongqing University, Chongqing, China; ^3^ Department of Information, The First Affiliated Hospital of Chongqing Medical University, Chongqing, China

**Keywords:** glioma, magnetic resonance imaging, deep learning, convolutional neural network, differentiation

## Abstract

**Background:**

Computational aid for diagnosis based on convolutional neural network (CNN) is promising to improve clinical diagnostic performance. Therefore, we applied pretrained CNN models in multiparametric magnetic resonance (MR) images to classify glioma mimicking encephalitis and encephalitis.

**Methods:**

A data set containing 3064 MRI brain images from 164 patients with a final diagnosis of glioma (n = 56) and encephalitis (n = 108) patients and divided into training and testing sets. We applied three MRI modalities [fluid attenuated inversion recovery (FLAIR), contrast enhanced-T1 weighted imaging (CE-T1WI) and T2 weighted imaging (T2WI)] as the input data to build three pretrained deep CNN models (Alexnet, ResNet-50, and Inception-v3), and then compared their classification performance with radiologists’ diagnostic performance. These models were evaluated by using the area under the receiver operator characteristic curve (AUC) of a five-fold cross-validation and the accuracy, sensitivity, specificity were analyzed.

**Results:**

The three pretrained CNN models all had AUC values over 0.9 with excellent performance. The highest classification accuracy of 97.57% was achieved by the Inception-v3 model based on the T2WI data. In addition, Inception-v3 performed statistically significantly better than the Alexnet architecture (*p*<0.05). For Inception-v3 and ResNet-50 models, T2WI offered the highest accuracy, followed by CE-T1WI and FLAIR. The performance of Inception-v3 and ResNet-50 had a significant difference with radiologists (*p*<0.05), but there was no significant difference between the results of the Alexnet and those of a more experienced radiologist (*p >*0.05).

**Conclusions:**

The pretrained CNN models can automatically and accurately classify these two diseases and further help to improving clinical diagnostic performance.

## Introduction

For an intracranial lesion, the first question faced by the neuroradiologist is whether it is a neoplastic or non-neoplastic lesion. Glioma and encephalitis are two common diseases of the central nervous system that sometimes overlap in their clinical symptoms and radiographic presentations (1). However, the treatment protocols and prognosis are substantially different for these two diseases. Being able to classify glioma and encephalitis both accurately and noninvasively is of the utmost importance.

MRI is most commonly used to assess brain diseases due to its superior contrast compared with other imaging modalities. In current conventional MR imaging methods, it is not difficult to classify encephalitis from a single enhancing glioma with perifocal edema, mass effect, and necrosis. However, some gliomas (referred to as “glioma mimicking encephalitis” in this paper, mainly lower-grade glioma) show focal area enhancement or no enhancement lesions without mass effect or necrosis, which may be misdiagnosed as encephalitis, resulting in delayed treatment (1, 2). On the other hand, some encephalitis have a certain mass effect due to the large scope, which may also be misdiagnosed as glioma for craniocerebral surgery or pathological biopsy (3).

Some advanced MR modalities such as diffusion-weighted imaging (DWI), MR spectroscopy (MRS) and perfusion-weighted imaging (PWI) play important roles in differentiating glioma and encephalitis to some extent. However, generally advanced imaging techniques require additional expense and time to perform and may not be routinely performed for every patient in clinical practice. By contrast, FLAIR, CE-T1WI, and T2WI are almost always available. However, conventional MRI modalities do not fully perform deep mining of the intrinsic features of images given the limitations of the subjective vision of the human eye. To improve the diagnostic accuracy and efficiency, advanced and automated methodologies are needed.

Recently, computational aid in diagnosis became a fast-developing research area, combining radiology imaging and computers in a noninvasive fashion to extract a large number of high-dimensional features to help improve clinical diagnostic performance (4, 5). Several studies on MRI brain tumour classification using traditional machine learning approaches such as the classification of glioblastoma (GBM) and primary cerebral nervous system lymphoma (PCNSL) used a support vector machine (SVM) or random forest (RF) (6–8). A few studies focused on the classification of a neoplastic or non-neoplastic lesion (9–11), such as autoimmune pancreatitis and pancreatic ductal adenocarcinoma. However, traditional machine learning methods have two main weaknesses. First, it depends on handcrafted features, which is time-consuming and highly dependent on the experience of the operators. Second, it focuses only on either low-level or high-level features (12). Deep learning is a subset of machine learning that does not require handcrafted features. Deep learning, especially convolutional neural networks (CNN), can achieve a higher classification performance than the traditional radiomic framework by automatically extracting the abstracted and deeper features from medical images (13, 14) and has been widely used for medical images analysis over the past several years (15, 16). Several successful studies have applied a pretrained CNN model such as Alexnet, ResNet, and GoogLeNet models to classify two or more types of tumours. To our knowledge, the application of deep learning based on multiparametric MRI to differentiate encephalitis from glioma mimicking encephalitis is rarely been reported.

Thus, in this study, we aimed to train CNN models to automatically classify glioma mimicking encephalitis and encephalitis by analyzing conventional multiparametric MRI images. We made three comparisons of the classification performance to find the most suitable classifier model for the classification problem: (a) a comparison of three existing pretrained CNN architectures (Alexnet, ResNet-50, and Inception-v3) with different parameters and layers, (b) a comparison of the effects of different MR modalities (FLAIR, CE-T1WI, and T2WI) on a model based on the same network, and (c) a comparison of quantified deep learning models with radiologists’ diagnostic performance.

## Materials and Methods

### Patients

For this retrospective analysis, ethical approval was obtained by our institutional review board (approval number 2019-178), and the informed consent requirement was waived. The study population consisted of 164 (56 gliomas and 108 encephalitis) patients enrolled at the institution consecutively between January 2012 and January 2020. The glioma mimicking encephalitis inclusion criteria were as follows: (1) histopathologically confirmed cerebral gliomas, and (2) patients with atypical MR imaging such as patchy or large patchy abnormal signals and insignificant enhancement without mass effect or necrosis, which are difficult to differentiate from encephalitis. Encephalitis inclusion criteria: encephalitis had the lesion on MRI, and was confirmed by cerebrospinal fluid analysis, antibody testing, virus examination, surgery or pathological biopsy, or confirmation of the diagnosis because the lesion completely or largely disappeared or turned into encephalomalacia during the follow-up period. In addition, before a routine MRI examination, no patients had a previous brain biopsy or treatment, and three MRI modalities (FLAIR, CE-T1WI, and T2WI) were used. The exclusion criteria were followed as: (1) the lesion was too small and its diameter was less than 10 mm, (2) MRI images had motion and other artifacts with poor quality that affected the analysis. The number of raw images in the glioma mimicking encephalitis and encephalitis group was 1570 and 1494 images, respectively.

### Data Acquisition

All MR images were obtained on a GE 3.0 T scanner (General Electric Medical Systems, USA) equipped with an eight-channel head coil. All images were stored in the picture archiving and communication systems (PACS, Carestream Health, Inc., Rochester, NY, USA). The acquisition parameters of were as follows: conventional axial T1WI [TR 250 ms, TE 2.86 ms, flip angle 90°, field of view (FOV) 240 × 240 mm, matrix 224 × 224, layer thickness 5 mm, total of 20 layers], axial T2WI [TR 3 600 ms, TE 120 ms, flip angle 90°, FOV 240 × 240 mm, matrix 256 × 256, layer thickness 5 mm, total of 20 layers] and FLAIR sequence [TR 8000 ms, TE 120 ms, flip angle 90°, FOV 240 × 240 mm, matrix 224 × 224, layer thickness 5 mm, total of 20 layers]. Axial contrast-enhanced T1WI (TR 6.3 ms, TE 3.1 ms, flip angle = 15°, FOV= 240 × 240 mm, matrix 192 ×192, slice thickness 5 mm) was obtained after intravenous rejection of 0.1 ml/kg gadobutrol (Gadovist, Bayer Schering Pharma). The scan range included the region from the calvarial vertex down to the foramen magnum.

### Neuroradiologist Assessment

To compare the diagnostic performance of the pretrained CNN models with a visual assessment, MRI images of all 164 cases were independently reviewed by the same two neuroradiologists (Wu JQ and Liu MQ, with 14 and 6 years’ experience, respectively, in neuroradiology). They were blinded to clinical information but were aware that the patients were either encephalitis or glioma without knowing the exact number of patients diagnosed with each entity. The two readers assessed only conventional MR images (FLAIR, CE-T1WI, and T2WI) and recorded a final diagnosis using a 4-point scale (1 = definite encephalitis; 2 = likely encephalitis; 3 = likely glioma; and 4 = definite glioma). Cohen’s kappa coefficients were used to assess the interdiagnosis agreement by the two radiologists, which was interpreted as follows: <0.20 = slight, 0.21–0.40 = fair, 0.41–0.60 = moderate, 0.61–0.80 = good, and 0.81–1.00 = excellent.

### Image Preprocessing

All MRI images (FLAIR, CE-T1WI, and T2WI) were exported in.jpg format without annotation from the original digital imaging communication in medicine (DICOM) format on PACS. Then we use the Opencv image-processing library to cut out the redundancy around the images, such as the skull and eyes, and only retain the brain parenchyma of the original image matrix. The flowchart was added to the supplementary materials. After that, images containing lesion areas were selected by the two experienced neuroradiologists. This screening can ensure that all features contributing to the classification are retained, and remove the nonfocus sections that interfere with the classification.

Data augmentation plays a vital role in the utilization of deep learning in medical images, especially in our task, which lacks data. The training data were augmented four times by randomly choosing four methods from a list of six methods: contrast transformation, brightness conversion (increased and decreased), sharpening and flipping (horizontal and vertical). The test data set was kept as origin. Last, in order to implement our experiment more efficiently, as the CNN’s network architecture we chose the bilinear interpolation to resize all the images to 224 × 224.

### Deep Transfer Learning

One of the most important reasons for the tremendous success of deep learning is that it can handle massive amounts of data. With the development of the Internet, thousands of different data can be obtained in a very short time. Nevertheless, in the field of medical imaging, there is usually a lack of data sets. Moreover, the annotation of medical images is not only tedious, laborious, and time-consuming but also demanding of costly, specialty-oriented skills that require experienced radiologists. Transfer learning is an effective method to solve this problem (17). Transfer learning is a method that uses a CNN network model trained on a large data set such as ImageNet and transfers it to another different but related task. This results in a faster, more accurate and more generalized learning process (18, 19). The weight parameters of this model are generated after learning massive data sets, and it has the ability to extract a class-specific feature representation, which is suitable for distinguishing encephalitis and glioma mimicking encephalitis.

**Figure 1:**
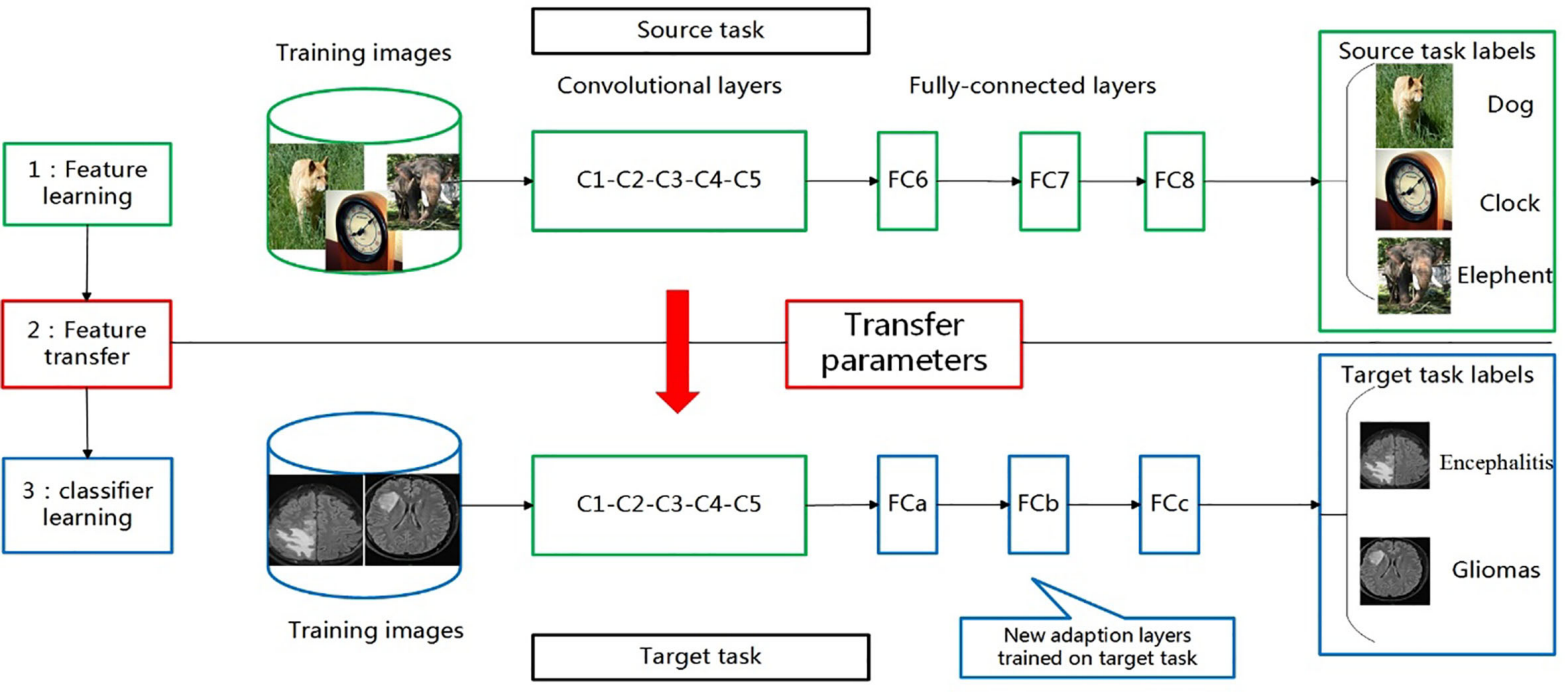
Transferring parameters of convolutional neural network (CNN). First, network is trained on source task (ImageNet classification, top row) with large amount of available labeled images. Then, convolutional layers (C1-C5) are transferred to target task(brain tumour and encephalitis classification, bottom row). We remove original fully connected layers (FC6-FC8) and add adaptation layer (FCa-FCc). Last, we fine-tune new model on labeled data of target task.

Based on the idea of transfer learning, we selected current popular CNN networks (Alexnet, ResNet-50, and Inception-v3) to classify encephalitis and glioma mimicking encephalitis of intracranial diseases with similar manifestations on MRI. We trained them using the ImageNet data set. After that, we utilized our own training data set to fine-tune these models and evaluate them in order to choose the model with the best performance. The transfer learning process for the classification of these two brain diseases is shown in **Figure 1**.

### Pretrained Model

After Alexnet was proposed and had tremendous success in image classification tasks during the ImageNet Large-Scale Visual Recognition Challenge (ILSVRC) in 2012, more and more deep convolutional neural networks with superior performance were proposed and applied in scientific research and practical applications, such as computer vision and natural language processing. All basic structures are the same, including the convolutional layer, pooling layer, and fully connected layer.

#### Alexnet

In 2012, the Alexnet was the first architecture to use a deep convolutional neural network, which showed the best results and achieved significant improvement over traditional non-deep methods for the ILSVRC task (20). It has several innovations including using ReLU as the CNN network’s activation function to solve the gradient dispersion problem when the network is deep, using the dropout method randomly to ignore some neurons and reduce overfitting and using max-pooling and proposed local response normalization (LRN) in the network to enhance the generalization ability. It has 60 million parameters and 500,000 neurons, and consists of five convolutional layers and three pooling layers (not shown) followed by three fully connected layers and a softmax classifier.

#### ResNet-50

The ResNet (21) model proposed a new structure called the “residual block,” which changed the learning goal: the residual block was no longer learning a complete network’s output, but the difference between output and input was residual. The residual block is implemented by a connection between the block’s input and an output named the “shortcut connection.” The input and output of this “residual block” are overlapped elementwise through a “shortcut connection,” which does not add additional parameters or computation to the network but can greatly accelerate the training process and improve the training effect of this model. By using a residual unit to successfully train 152 layers of the deep neural network, this approach was the champion in the ILSVRC 2015 competition, achieving a 3.57% top-5 error rate while the parameter quantity was lower than those of other deeper models. Because of its “simple and practical” coexistence, after that, many methods based on ResNet-50 or ResNet-101 have been widely used in detection, segmentation, recognition, and other fields.

In our task, we chose a ResNet-50 model containing one general convolutional layer, 16 “building block” modules and one pooling layer and one fully connected layer. Each “building block” has three convolutional layers. ResNet-50 eventually has 20 million parameters.

#### Inception-v3

The GoogLeNet (22) model is significantly more complex and deeper than all previous CNN architectures. More important, it introduces a new module called the “inception module,” which concatenates filters of different sizes and dimensions into a single new filter. In our task, we chose the Inception-v3 architecture to train a classifier. Overall, Inception-v3 has six convolutional layers, two pooling layers, and ten “inception” modules. Each “inception” module consists of seven to nine convolution layers and one pooling layer. The model we chose contains 30 million parameters.

### Architecture Modification

The models we introduced above were for ILSVRC image classification tasks that contain 1000 categories. However, in our task, there are only two categories, so the original CNN structures need to be modified to ensure that the experiment can be implemented. First, because of a lack of data sets and a large number of parameters in these models, overfitting occurs, which means that the training accuracy is outstanding but the validation or test accuracy is much lower. This suggests that the model is too complex to fit the data and the data are too scarce. To alleviate this problem, we removed all fully connected layers and treated the activations of the last convolutional layer as a deep feature representation for each input image. We added two fully connected layers which contained fewer hidden nodes to reduce the parameters. The first fully connected layer was fixed at 512 hidden nodes, and we initialized the weights with a Gaussian distribution. The activation function was ReLU. The second fully connected layer had 256 hidden nodes, and its initialization and activation were the same as that of the first fully connected layer. The last layer was the output layer. For our binary classification task, we fixed the last layer at one. We used sigmoid activation at the output layer to determine the probability directly. We used other modifications on the models to promote their classification performance, such as adding batch normalization and dropout. All of these modifications are based on regularization, which can alleviate overfitting and increase the classification accuracy.

### Experiment

#### Evaluation

Because we had a small amount of data, the evaluation of the three models was based on five-fold cross-validation, which splits all samples into five subfolds, using four of them as a training set and one as a test set in each iteration. During the experiment, the training process was carried out on the training set, while the test set was used to assess the performance of each model.

Due to the imbalances between the two diseases in our task, we used the receiver operating characteristic (ROC) curve that is drawn by the false positive rate and true positive rate as our principle evaluation measure. The ROC and the area under the curve (AUC) were used to evaluate the discrimination performance of these models using DeLong tests, and *p*<0.05 was considered statistically significant. The accuracy, sensitivity, and specificity were also computed.

#### Implementation

The preprocessing and classification methods were coded in python using Keras and Opencv. Evaluation methods were implemented using scikit-learn. All experiments were performed using Ubuntu OS on a machine with an Intel Xeon E5 2687W V3, NVIDIA GeForce 1080ti GPU, and 16 × 8GB of RAM.

The CNN networks we chose (Alexnet, ResNet-50, and Inception-v3) were all fine-tuned from ImageNet pretrained models. In these experiments, we fixed the hyperparameters to be the same so that we could choose the best model with excellent performance. For fine-tuning, the number of training epochs was 50, and the minibatch size was 32 image instances. We used Adam as our optimization method, and the hyperparameters had momentum: 0.9, weight decay: 0.0005 and base-learning rate: 0.001.

## Results

### Comparison of Performance of Three Pretrained CNN Models

**Figure 2:**
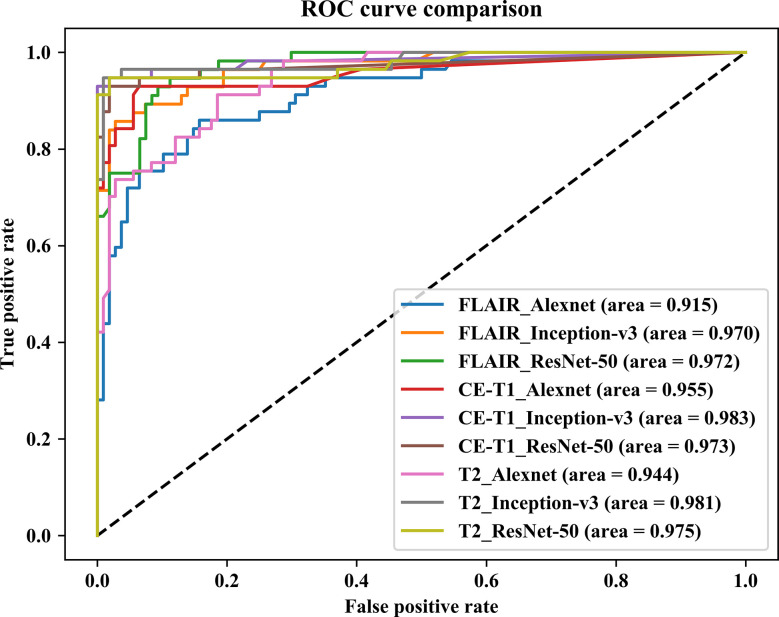
Comparison of receiver operating characteristic curves of three pretrained CNN models based on three single MRI modalities.

In this paper, we focused on three pretrained CNN models in classifying glioma mimicking encephalitis and encephalitis. **Table 1** summarizes the averaged classification accuracy, specificity, sensitivity and AUC values of the Alexnet, ResNet-50, and Inception-v3 architectures based on three single MRI modalities under five-fold cross-validation. **Figure 2** shows the ROC curves of the three pretrained CNN methods based on three single MRI modalities. It can be seen from **Table 1** and **Figure 2** that all classification models had great potential in distinguishing glioma mimicking encephalitis and encephalitis (AUC>0.9). Among them, the highest classification performance was achieved by the Inception-v3 model, and the lowest performance was obtained with the Alexnet model. And Inception-v3 performed statistically significantly better than the Alexnet architecture in accuracy (*p*<0.05). The classification accuracy on FLAIR, CE-T1WI and T2WI of ResNet-50 was 88.95%, 93.33% and 95.75%, respectively. The Alexnet performance was statistically significantly lower than that of the ResNet-50 architecture when based on FLAIR modality (*p*<0.05). There was no statistically significant difference between ResNet-50 and Inception-v3 for any of the modalities.

**Table 1:** Performance comparison of three pretrained models based on three single MRI modalities.

		Accuracy (%)	Specificity (%)	Sensitivity (%)	AUC
**FLAIR**	**Alexnet**	86.67	95.37	70.17	0.915
	**ResNet-50**	88.95	92.52	82.14	0.972
	**Inception-v3**	93.29	97.22	85.71	0.970
**CE-T1WI**	**Alexnet**	92.72	97.22	84.21	0.955
	**ResNet-50**	93.33	100	80.70	0.973
	**Inception-v3**	96.96	100	91.22	0.983
**T2WI**	**Alexnet**	84.84	87.96	78.94	0.944
	**ResNet-50**	95.75	96.29	94.73	0.975
	**Inception-v3**	97.57	99.07	94.73	0.981

AUC, the area under the receiver operator characteristic curve.

### Comparison of Performance of Single Modality Model

**Figure 3:**
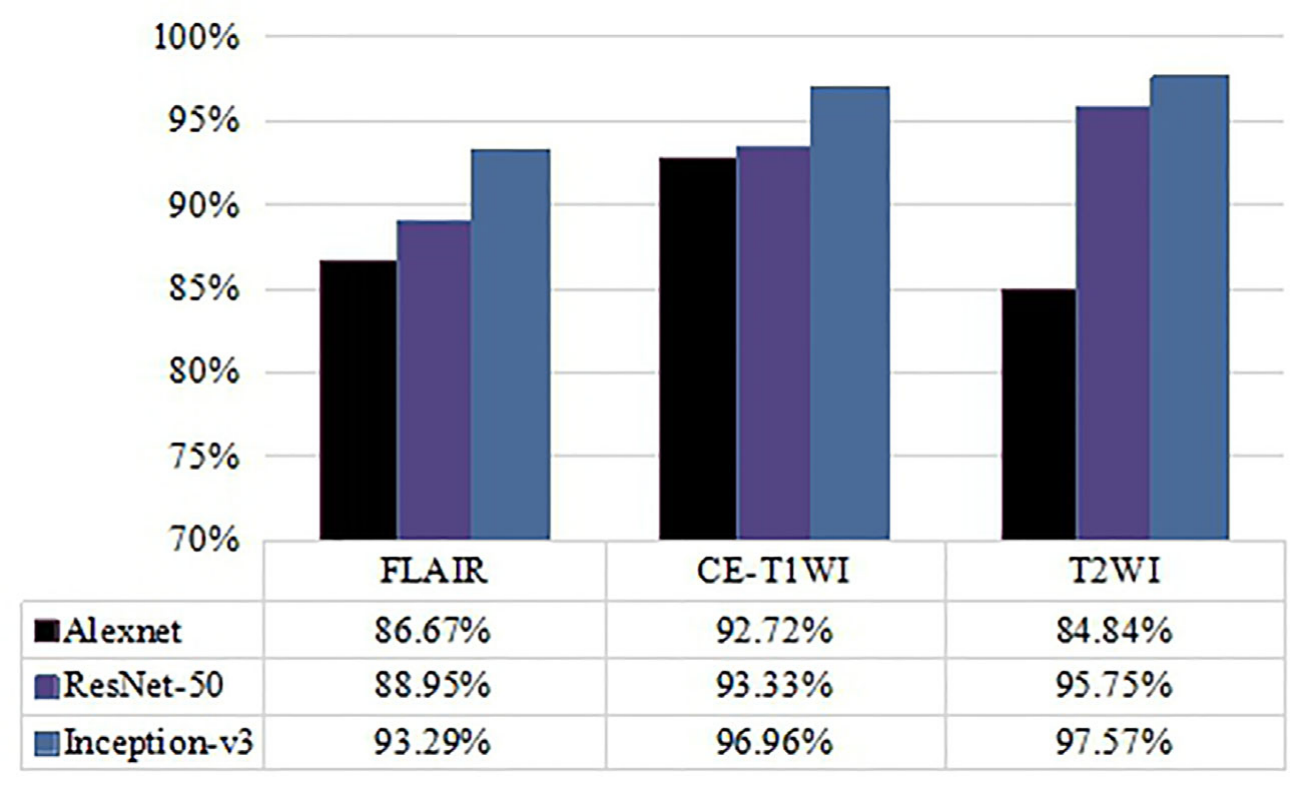
Comparison of accuracy of classification performance with Alexnet, ResNet-50, and Inception-v3 on three single MRI modalities.

To further examine the effect of different MRI modalities on model performance, FLAIR, CE-T1WI, and T2WI single modality networks were constructed, and the classification accuracies are shown in **Figure 3**. The results indicate that the Inception-v3 and ResNet-50 models related to T2WI data inputs achieved the highest accuracy (97.57% and 95.75%, respectively) and were slightly better than CE-T1WI and FLAIR data. However, they had similar AUC values on T2WI and CE-T1WI. Looking at the classification performance of the Alexnet model, the highest accuracy (92.72%) and AUC (0.955) were achieved based on CE-T1WI. FLAIR had the lowest classification ability for the three networks. Note that no matter what network was applied, the three single MRI modalities (FLAIR, CE-T1WI, and T2WI) as the input data had no significant difference in their classification performance (*p*>0.05, DeLong test).

### Comparisons of Visual Assessment and Deep Learning Methods

**Figure 4:**
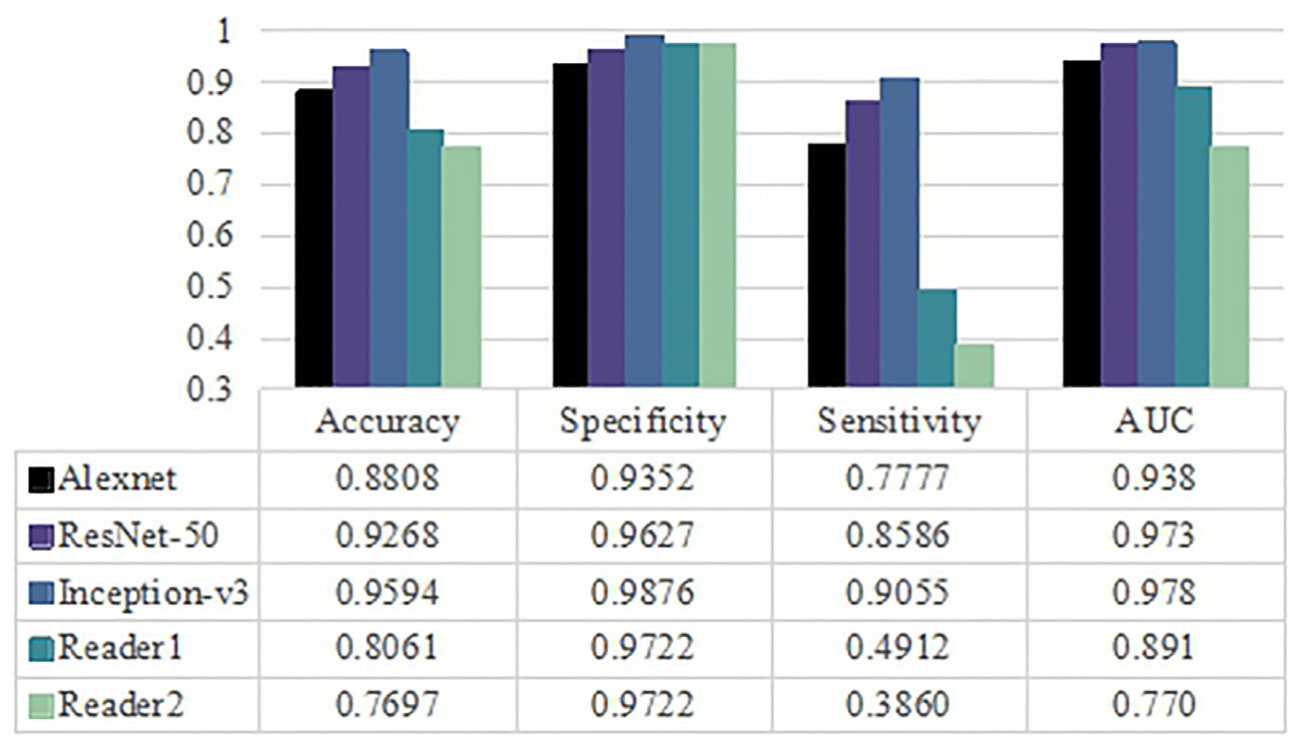
Classification performance for three pretrained models and radiologists including averaged accuracy, specificity, sensitivity and AUC value as evaluation metrics.

The diagnostic performance of visual assessment for these two diseases was evaluated by two neuroradiologists. The AUC were 0.891, 0.770 and the accuracy were 80.61% and 76.97% for readers 1 and 2, respectively. Interagreement between the two neuroradiologists was rated as moderate (Cohen’s kappa = 0.513, 95% CI was 0.415–0.611). Based on the results in **Figure 4**, the Inception-v3 and ResNet-50 models had significant differences from the results of two neuroradiologists with regard to AUC (*p*<0.05, DeLong test) for any MRI modality. However, there was no significant difference between the AUC of the Alexnet model and those of the experienced neuroradiologist of reader 2 (*p*>0.05, DeLong test). These findings indicate the Inception-v3 and ResNet-50 models have a higher level of performance than radiologists' diagnostic performance. The performance of the Alexnet model is better than that of a resident neuroradiologist and equivalent to that of an experienced neuroradiologist.

## Discussion

In this study, three pretrained CNN models were fine-tuned using a transfer learning approach that was successfully implemented for the automated classification of glioma mimicking encephalitis and encephalitis on conventional MR images. Classification models with AUC values of 1.00–0.90 and 0.90–0.80 were regarded as excellent and good, respectively. In this study, no matter what MRI modality as the input data, three pretrained CNN network all had AUC values over 0.9 with excellent performance. Inception-v3 based on the T2WI modality achieved the highest classification accuracy. And then we demonstrated the utility of deep learning to classify neoplastic and non-neoplastic situations of the brain, yielding excellent diagnostic values over the visual analysis of neuroradiologists, which is consistent the results of other studies (6–8, 23).

In terms of the structure of the three pretrained CNN models, namely Alexnet, ResNet-50, and Inception-v3 models for our classification problem. The Inception module of the Inception-v3 was able to extract features under different receptive fields, and combine these features to obtain deep features with stronger robustness, so as to achieve the best classification accuracy in this study. The structure of ResNet-50 model mainly improved the perspective of model optimization, but the size of receptive field was unchanged, resulting in its performance lower than Inception-v3. Notably, there was no significant difference between Inception-v3 and ResNet-50 models. As for Alexnet model, the number of convolution layers was too small to obtain advanced convolution features, resulting in the worst classification performance, which was significantly lower than that of inception-v3 and slightly lower than that of ResNet-50. Therefore, the deeper-layered Inception-v3 and ResNet-50 models provided features that are more suitable for distinguishing these two diseases than the thin-layered Alexnet model. Previous studies also confirmed it. Yang et al. (24) used Alexnet and GoogLeNet in grading glioma from MR images. GoogLeNet proved superior to Alexnet for the task. Talo et al. (15) demonstrated that the ResNet-50 model achieved the best classification accuracy while the Alexnet model obtained the lowest performance among Alexnet, ResNet-18, ResNet-34, and ResNet-50 pretrained models in five classes of brain abnormality classification MR images. In another study (25), the ResNet-50 model also achieved the highest classification accuracy among four pretrained models in automating four classes of oral squamous cell carcinoma.

Selecting which images to use is usually the basis for a machine learning study. In fact, the different MR imaging modalities used in machine learning studies of glioma in the current literatures with various conclusions. Some researchers reported that radiomics features extracted from the CE-T1WI with a better performance than other single MR sequences when grading the gliomas (26, 27) and predicting the IDH genotype of glioma (28). Another report showed that T2WI modality had the best IDH genotype prediction ability of glioma, and conversely, CE-T1WI was lower (29). In this study, as **Table 1** revealed, no matter what network was applied, the single MRI modality obtained high and similar performance. Among them the Inception-v3 and ResNet-50 diagnostic models based only on the T2WI modality conferred a slightly higher accuracy, followed by CE-T1WI and FLAIR. This probably because the patients who we chose in this study without significant enhancement, making it was difficult to determine the boundaries of glioma mimicking encephalitis and encephalitis lesions on the CE-T1WI modality, when we used entire MRI image rather than the ROI of the lesion. On the contrary, glioma mimicking encephalitis and encephalitis showed hyperintense signal intensities on T2WI modality, and hyperintense signal intensity on T2WI modality is also a hallmark of encephalitis (30). Therefore, the pretrained CNN models are easy to identify lesions and extract deep features on the T2WI modality. It is worth noting several studies showed that a combination of several MRI parameters can better understand the tumour characteristics with an enhanced performance of the classifier than a single modality model based on machine learning (27–29). However, Yoganada et al. (31) suggested that the T2WI network and multicontrast network achieved similar IDH classification accuracies of gliomas using deep learning MRI networks. Chang et al. (28) showed that CE-T1WI images achieved similar accuracy with a combined sequence model in a predictive glioma IDH genotype. This suggests that the information from single MR images can also provide a high classification confidence, which is consistent with our results. Moreover, the ability to utilize only single MR modality data will facilitate imminent clinical translation.

There are some limitations in the present study. First, the sample size was relatively small for deep learning analysis and single-center study. Therefore, multicenter data sets and a larger patient cohort are needed to verify the current findings. Second, we did not include clinical data and imaging features to train a clinical model to compare with CNN models, and we will continue to study in the future. Third, we did not evaluate the diagnostic performance of deep learning based on T1WI sequence as it is difficult to automatically identify lesions, besides, the diagnostic performance of deep learning was not compared with combined sequences since the single sequence has achieved well results.

## Conclusions

In this study, we applied the transfer learning approach of three pretrained CNN models (Alexnet, ResNet-50, and Inception-v3) to automatically classify glioma mimicking encephalitis and encephalitis on conventional MRI images. The results demonstrated that the three pretrained CNN models had excellent classification performances that were superior to those of the neuroradiologists. The Inception-v3 and ResNet-50 were significantly superior to Alexnet. And no matter what network was applied, the single MRI modality as the input data can obtain high and similar performance, among them Inception-v3 related to T2WI input achieved the highest classification accuracy. Thus, the pretrained CNN models can aid in the accurate and noninvasive classification of glioma mimicking encephalitis and encephalitis by automatically extracting deep features from multiparametric MRI images.

## Data Availability Statement

The original contributions presented in the study are included in the article/**Supplementary Material**. Further inquiries can be directed to the corresponding author.

## Ethics Statement

The study involving human participants was reviewed and approved by The First Affiliated Hospital of Chongqing Medical University. For this retrospective analysis, the informed consent requirement was waived.

## Author Contributions

Conception and design: SX and WW. Provision of study material or patients: WW, WZ, and QW. Collection and/or assembly of data: WW, WZ, and QW. Data analysis and interpretation: WW, JL, and JY. Manuscript writing: WW, JL, and WZ. Manuscript review: SX and JY. Final approval of manuscript: WW, JL, JY, QW, WZ, and SX. All authors contributed to the article and approved the submitted version.

## Funding

This research was supported by Chongqing Research Program of Basic Research and Frontier Technology [no. cstc2018jcyjAX0633] and the Fundamental Research Funds for the Central Universities [no. 2018CDXYGD0017]. We appreciate the funding to support the research.

## Conflict of Interest

The authors declare that the research was conducted in the absence of any commercial or financial relationships that could be construed as a potential conflict of interest.
