# Supplementary figures and images for: Differentiation of Glioma Mimicking Encephalitis and Encephalitis Using Multiparametric MR-Based Deep Learning
Source: Front Oncol. 2021 Mar 15;11:639062. doi: 10.3389/fonc.2021.639062 (PMC8005708; doi:10.3389/fonc.2021.639062)

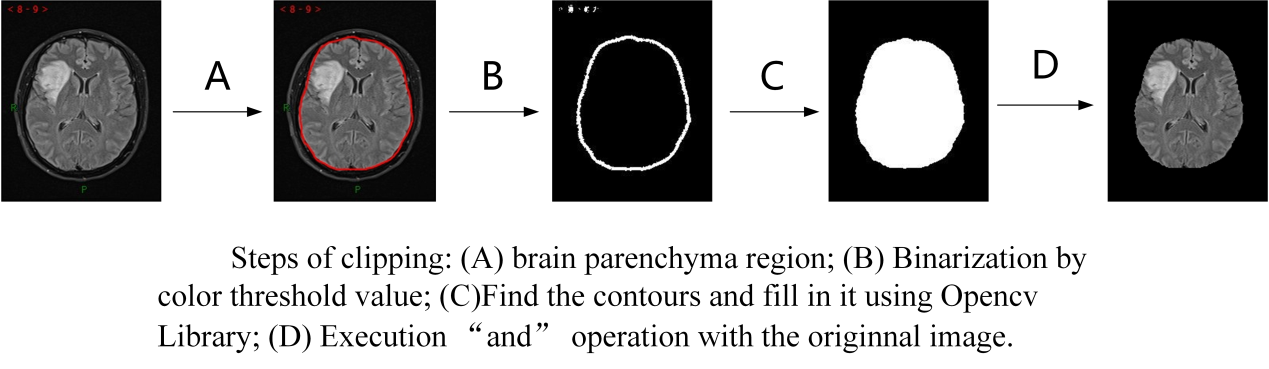

Supplement: Supplementary file 1 [file Table_1.docx]
